# Immunoaffinity Intact-Mass Spectrometry for the Detection of Endogenous Concentrations of the Acetylated Protein Tumor Biomarker Neuron Specific Enolase

**DOI:** 10.1021/acs.jproteome.4c00391

**Published:** 2024-07-16

**Authors:** Sebastian A. H. van den Wildenberg, Sylvia A. A. M. Genet, Maarten A. C. Broeren, Joost L. J. van Dongen, Luc Brunsveld, Volkher Scharnhorst, Daan van de Kerkhof

**Affiliations:** † Laboratory of Chemical Biology, Department of Biomedical Engineering, Eindhoven University of Technology, 5612 AZ Eindhoven, The Netherlands; ‡ Clinical Laboratory, Catharina Hospital, 5623 EJ Eindhoven, The Netherlands; § Expert Center Clinical Chemistry Eindhoven, 5612 AZ Eindhoven, The Netherlands; ∥ Clinical Laboratory, Máxima Medical Center, 5504 DB Veldhoven, The Netherlands

## Abstract

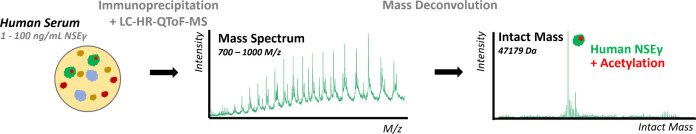

Intact-mass spectrometry has huge potential for clinical application, as it enables both quantitative and qualitative analysis of intact proteins and possibly unlocks additional pathophysiological information via, e.g., detection of specific post-translational modifications (PTMs). Such valuable and clinically useful selectivity is typically lost during conventional bottom-up mass spectrometry. We demonstrate an innovative immunoprecipitation protein enrichment assay coupled to ultrahigh performance liquid chromatography quadrupole time-of-flight high resolution mass spectrometry (UPLC-QToF-HRMS) for the fast and simple identification of the protein tumor marker Neuron Specific Enolase Gamma (NSEγ) at low endogenous concentrations in human serum. Additionally, using the combination of immunoaffinity purification with intact mass spectrometry, the presence of NSEγ in an acetylated form in human serum was detected. This highlights the unique potential of immunoaffinity intact mass spectrometry in clinical diagnostics.

## Introduction

The use of mass spectrometry as an alternative to immunoassay-based methods for the quantification of proteins is of great interest in the field of clinical chemistry. Commonly, bottom-up strategies are used in which proteins are digested into proteotypic peptides. One, or a limited number, of the obtained peptides are then selected as representative for the whole protein and subsequently quantified using selective MS/MS fragmentation based methods using quadrupole mass analyzers, similar to small molecules.^1,2^ Signature peptides need to be carefully chosen to enable selective quantification of protein isoforms or isozymes.^3−5^ The peptides carrying a post-translational modification (PTM) are mostly avoided, as this complicates the reproducible quantification and the production of suitable reference materials and stable isotope labeled (SIL) peptide internal standards. The downside of this signature peptide selection is that valuable information about the protein is thus lost, as the presence of PTMs may carry valuable pathophysiological information.^6,7^ Different from bottom-up and middle-down based methods, top-down mass spectrometry analyzes the whole protein, retaining selectivity both for different isozymes and for PTMs.^8−11^ Additionally, sample preparation is simplified by removing the protein digestion step. However, intact mass spectrometry requires high-resolution MS (HRMS), such as Time-of-Flight, FT-ICR or Orbitrap mass analyzers. These instruments are not yet widely available in (routine) clinical laboratories.^12,13^ Sensitivity is also relatively low because of the multiple charge states of large proteins and matrix effects caused by ion suppression.^14^ Finally, when the intact mass spectrometry methodology is to be applied in clinical diagnostics, the required fully characterized and commutable reference materials and full-protein-labeled internal standards pose a significant challenge due to the complexity of the protein analytes. As such, there is a strong need for successful case studies detecting full length protein biomarkers at physiological concentrations from human serum.

Neuron-Specific Enolase (NSE) is a biomarker composed of αγ- and γγ-dimers that is used for both diagnosis and follow-up in lung cancer.^15^ Using bottom-up LC-MS assays, it is already possible to distinguish between these isoforms, as was demonstrated in previous work.^4^ However, the development of an intact mass proteomics method could lead to a simplified method for isoform differentiation, incorporation of PTMs in the analysis, and quantification and correction for, e.g., hemolysis, since erythrocytes contain high concentration of αγ-NSE.^16^

In this study, the lung cancer biomarker NSEγ was therefore analyzed in human serum via immunoaffinity purification coupled to intact mass spectrometry. Anti-human NSEγ antibodies were coupled and cross-linked to protein G labeled Dynabeads for the immunoprecipitation of NSEγ from human serum. Subsequently, the protein of interest was eluted from the magnetic-bead-antibody complex and analyzed by using liquid chromatography coupled to high resolution quadrupole time-of-flight mass spectrometry (LC-QToF-HRMS).

## Experimental Section

Antibody coupling to protein G functionalized magnetic Dynabeads was performed as published previously.^17^ Briefly, 2 μg of anti-human NSE 9601 SPTN-5 monoclonal mouse antibody (LOT: 0046841, Medix Biochemica, Espoo, Finland) (anti-NSEγ antibody) was coupled to 0.25 mg of Protein G Dynabeads (Thermo Fisher Scientific, Waltham, MA, USA) to obtain one equivalent, suitable for one isolation experiment. BS(PEG)_5_ (Sigma-Aldrich, Saint Louis, MO, USA) cross-linking of the protein G and antibody was performed at a concentration of 25 μM. One equivalent of coupled beads (50 μL) was incubated in 1.0 mL of serum sample or the NSEγ-depleted serum with rotation for 2 h. The beads were successively washed two times with 200 μL of PBS, pH 7.4. After the first wash, the bead suspension was transferred to a new LoBind Eppendorf tube. The beads were then resuspended in 50 μL of elution buffer (Milli-Q water/acetonitrile (ACN) (80:20) + 1.0% formic acid (FA)) and incubated for 5 min. Then, the beads were removed, and the supernatant was transferred to LC-vials for UPLC-QToF-HRMS analysis. Analysis of the samples was performed using a Xevo G2-XS HR QToF coupled to an Acquity UPLC I-class binary solvent manager and Acuity UPLC Sample Manager-FL (Waters, Milford, MA, USA). A Thermo Scientific MAbPac reversed phase HPLC column (4 μm, 2.1 mm × 50 mm) (Waltham, MA, USA) was used for chromatography at a column temperature of 80 °C. Flow rate was set at 0.3 mL/min, and a gradient of Milli-Q containing 0.1% (v/v) FA (A) and ACN containing 0.1% (v/v) FA (B) was set as follows (all displayed as % v/v): 0.0–11.5 min (25–50% B), 11.5–12.5 min (50–75% B), 12.5–13.0 min (75% B), 13.0–13.1 min (75–25% B), 13.1–15.0 min (25% B). Electrospray ionization (ESI) was operated in positive ionization mode. Mass Spectrometry settings were set as follows: capillary voltage: 0.8 kV; sampling cone: 40; source offset: 80; source temperature: 120 °C; desolvation temperature: 450 °C; cone gas: 10 L/h; and desolvation gas: 1000 L/h. Prior to the batch analysis, the instrument was calibrated using phosphoric acid over a range of 100 to 2000 *m*/*z*. LeuEnk (556.27 *m*/*z*) was used as LockSpray during each measurement at 0.5 min intervals. Extracted Ion Chromatograms (XIC) were created by isolating the 5 most abundant charge states, 814.42, 828.68, 843.46, 858.77, and 874.70 *m/z* (58+ to 54+), with a window of 0.1 Da. Deconvolution was performed by selecting the 5 most abundant charge states, corresponding to the ones selected for XIC. The Waters Maximum Entropy based tool for interpreting multiply charged electrospray data (MaxEnt 1) was used to achieve the artifact-free zero-charge spectrum based on the detected charged states of NSE.^18^ MaxEnt 1 deconvolution was performed over a range of 46000 to 49000 Da, with a resolution of 0.10 Da/channel. A simulated isotope pattern was used with a spectrometer blur width of 0.330 Da as the damage model. Left and right minimum intensity ratios were set at 33%. Completion was set to iterate to convergence. Further, more peaks were centered to determine the MaxEnt error. The theoretical NSEγ-mass was accurately calculated using the atomic weights of common elements in proteins (land plants with the C3 metabolic process and other organic sources): C: 12.01079, H: 1.007968, N:14.00669, O:15.99937 and S: 32.0639 Da.^19^

## Results and Discussion

**Figure 1:**
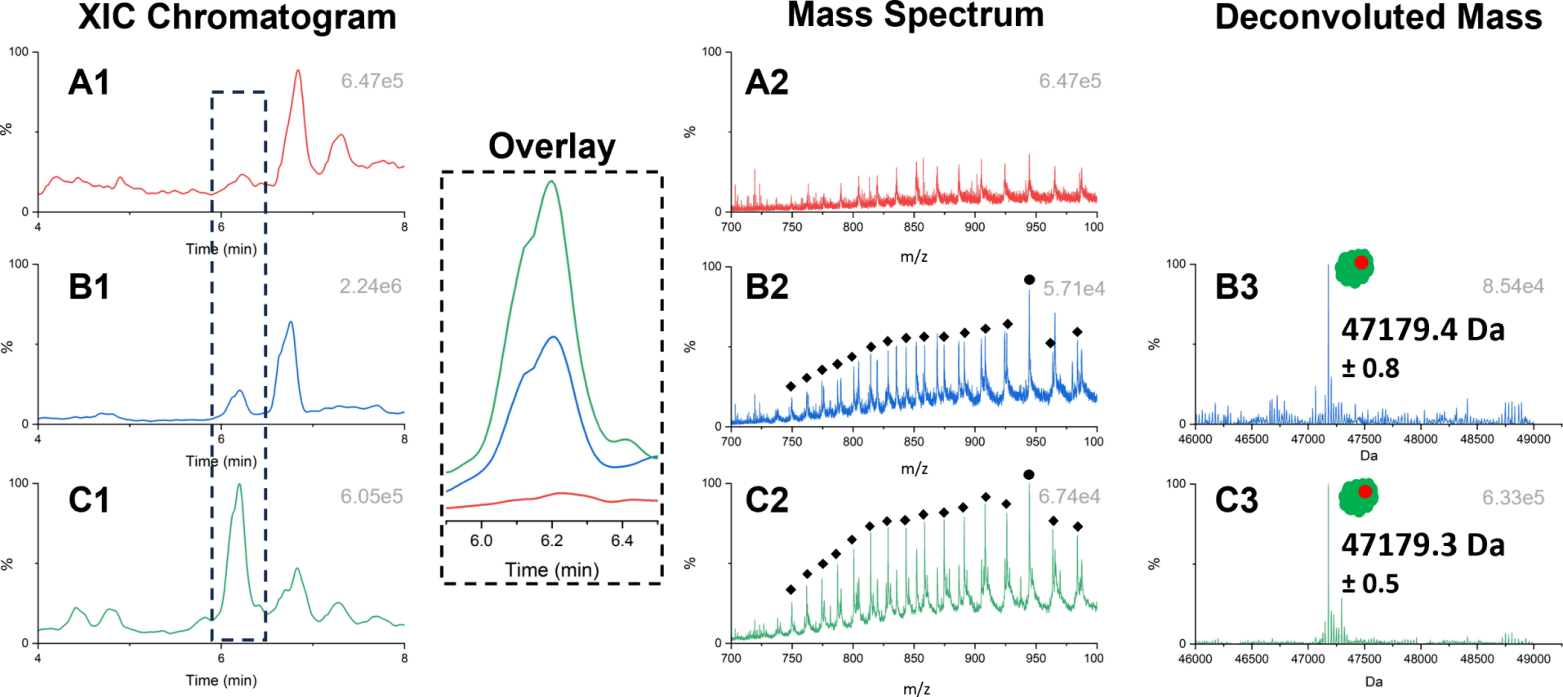
LC-HRMS analysis after NSEγ-immunoprecipitation. Extracted Ion Chromatogram (XIC) of NSEγ-depleted serum (A1) and sera with low (B1) and high (C1) endogenous NSEγ concentration, with combined overlay (Overlay). Mass spectra of NSEγ-depleted serum (A2) and sera with 16.0 ng/mL (B2) and 73.5 ng/mL (C2) NSEγ concentration. NSEγ related peaks are indicated with diamonds (◆). MaxEnt 1 deconvoluted mass of sera with 16.0 ng/mL (B3) and 73.5 ng/mL (C3) NSEγ concentration with observed acetylation.

NSEγ correlating to the presence of NSEαγ- and γγ-dimers was successfully isolated from human serum using immunoprecipitation (IE) coupled to LC-QToF-HRMS. Based on results of an alternative quantification method (ECLIA assay, Roche Diagnostics, Rotkreutz, Switzerland), a blank (NSE-depleted serum, <LLoQ) and 16.0 ng/mL and 73.5 ng/mL concentration samples were selected to demonstrate the adequacy of the method at different concentration levels. Anonymized left-over serum was used as sample material. The blank plasma was produced by repeated immunopurification from random serum with normal NSE concentration. The results of the reconstructed extracted ion chromatograms (XICs) are displayed in the XIC-panel in Figure 1. As expected, no NSEγ was detected in the depleted serum (A1), making it suitable as a blank reference material for comparison. NSEγ was easily detected from human sera, at both low (B1) and high (C1) concentration. Full scan chromatograms and the spectra of other observed peaks can be found in Figures S1–S5.

In the mass spectrum panel of Figure 1, the MS-spectra between 6.0 and 6.3 min are displayed. No NSEγ related peaks were observed in the NSE Depleted Serum (NDS) sample, confirming the absence of NSEγ (A2). Clear NSEγ charge state envelopes were observed in both the 16.0 ng/mL (B2) and 73.5 ng/mL serum samples (C2). All NSEγ related peaks are indicated with diamonds (◆). The peak in the mass spectra indicated by the circle (●) is a result of the 50+ charge state peak of NSEγ combined with the signal of an unknown background protein. The 50+ charge state is not used for mass deconvolution, since the charge states 58+ to 54+ are used. Additionally, in all three samples, noise peaks were observed. These peaks were found to be non-NSE related and did not influence the final mass deconvolution.

From these charge state envelopes, five charge states could easily be selected to perform MaxEnt 1 deconvolution to determine the intact full mass of the protein. In both the 16.0 ng/mL (B3) and 73.5 ng/mL (C3) concentration samples, intact masses of 47179.38 (±0.83) and 47179.33 (±0.47) Da were determined, respectively. These intact masses differ by +42 Da from the calculated theoretical average mass of 47137.07 Da based on the amino acid sequence of NSEγ (Supporting Material S1). These mass differences indicate a single protein acetylation event. This is, to the best of our knowledge, the first low throughput screening, HR-MS observation of NSEγ acetylation. Conclusions drawn from various high throughput screening studies were not in agreement for a specific acetylation site, with K197, K199 and K233 being reported.^20,21^ No literature was found on NSE trimethylation, the only possible other source for a +42 Da mass shift, therefore suggesting that the mass shift is caused by acetylation. Additionally, and in contrast to these conflicting studies, we observed only one singular acetylation event. Furthermore, no unacetylated-NSEγ or other (multiple acetylated) NSEγ isoforms were detected, with an estimated limit of detection around 5 ng/mL.

With this first example on NSEγ, this study demonstrates the power of coupling immunoprecipitation purification to LC-QToF-HRMS for full-length detection of low concentration protein tumor markers directly from human serum and additionally detecting a PTM that is lost during bottom-up detection of NSEγ. The concomitant identification of an acetylation PTM for this low abundance serum protein tumor biomarker at both high and low endogenous concentrations further testifies to the huge potential of this approach for both analytical and clinical purposes. Intact-mass proteomics can simultaneously detect low-concentration biomarkers and provide valuable extra information compared to conventional bottom-up MS and immunoassays that are currently in use: a substantial advancement in the field of targeted proteomics. Current work is focusing on the analytical validation of the method described in this Letter. This work serves as a springboard for the analysis of other proteins and the discovery of other clinically relevant proteoforms. The distribution and abundance of these families of pro-reform members would be expected to have a clinical relevance. For further clinical use, fully characterized and commutable reference materials and internal standards are required. Interestingly, reference proteins without the endogenously present PTM might be suitable as reference material. Additionally, future work should focus on the quantification of NSEγ to make it suitable for use in the clinical laboratory, include the detection of NSEα in a multiplex fashion, and determine if any PTM or amino acid variation within the binding epitope of the protein could compromise the measurements.

## Data Availability

The mass spectrometry raw data has been deposited in ProteomeXchange/PRIDE^22^ archive database, identifier: PXD053170.

## Supporting Information Available

Figure S1: Chromatogram of NSEγ-depleted serum (A1) and sera with low (B1) and high (C1) NSEγ concentration after immunoprecipitation, with the retention time of NSEγ indicated in the dashed-line box; Figure S2: Chromatogram (A1), charge state envelope (A2) and deconvoluted intact mass (A3) of co-immunoprecipitating protein at tr: 3.58 min; Figure S3: Chromatogram (A1), charge state envelope (A2) and deconvoluted intact mass (A3) of co-immunoprecipitating protein at tr: 4.20 min; Figure S4: Chromatogram (A1), charge state envelope (A2) and deconvoluted intact mass (A3) of co-immunoprecipitating protein at tr: 4.83 min; Figure S5: Chromatogram (A1), charge state envelope (A2) and deconvoluted intact mass (A3) of co-immunoprecipitating protein at tr: 6.76 min; Supp Material S1: Sequence of Neuron Specific Enolase gamma obtained from Uniprot (P09104) (PDF)
