# Supplementary material for: Immunoaffinity Intact-Mass Spectrometry for the Detection of Endogenous Concentrations of the Acetylated Protein Tumor Biomarker Neuron Specific Enolase
Source: J Proteome Res. 2024 Jul 16;23(8):3726–30. doi: 10.1021/acs.jproteome.4c00391 (PMC11301673; doi:10.1021/acs.jproteome.4c00391)
Supplement: Supplementary file 1 — pr4c00391_si_001.pdf [file pr4c00391_si_001.pdf]

Immunoaffinity Intact-Mass Spectrometry for the Detection of Endogenous Concentrations of the Acetylated Protein Tumor Biomarker Neuron Specific Enolase

Sebastian A. H. van den Wildenberg[1, 2, 3], Sylvia A. A. M. Genet[1, 2, 3], Maarten A. C. Broeren[1, 3, 4], Joost L. J. van Dongen[1, 3], Luc Brunsveld[1, 3], Volkher Scharnhorst[1, 2, 3], Daan van de Kerkhof[1, 2, 3,*].

1 Laboratory of Chemical Biology, Department of Biomedical Engineering, Eindhoven University of Technology, 5612 AZ, Eindhoven, the Netherlands

2 Clinical Laboratory, Catharina Hospital Eindhoven, 5623 EJ, Eindhoven, the Netherlands

3 Expert Center Clinical Chemistry Eindhoven, 5612 AZ, Eindhoven, the Netherlands

4 Clinical Laboratory, Máxima Medical Center, Eindhoven/Veldhoven, 5504 DB, Veldhoven, the Netherlands.

* Corresponding author: Daan van de Kerkhof: daan.vd.kerkhof@catharinaziekenhuis.nl

Supporting Information Table of Contents

1. **Figure S1**

   Chromatogram of NSEγ-depleted serum (A1), sera with low (B1) and high (C1) NSEγ concentration after immunoprecipitation

2. **Figure S2**

   Chromatogram (A1) Charge state envelope (A2) and deconvoluted intact mass (A3) of co-immunoprecipitating protein at tr: 3.58 min.

3. **Figure S3**

   Chromatogram (A1) Charge state envelope (A2) and deconvoluted intact mass (A3) of co-immunoprecipitating protein at tr: 4.20 min.

4. **Figure S4**

   Chromatogram (A1) Charge state envelope (A2) and deconvoluted intact mass (A3) of co-immunoprecipitating protein at tr: 4.83 min and is expected to be Humans Serum Albumin.

5. **Figure S5**

   Chromatogram (A1) Charge state envelope (A2) and deconvoluted intact mass (A3) of co-immunoprecipitating protein at tr: 6.76 min and is expected to be Apolipoprotein AI.

6. **Supporting Material S1**

   Amino Acid Sequence Neuron Specific Enolase Gamma

Figure S1: *Chromatogram of NSEγ-depleted serum (A1), sera with low (B1) and high (C1) NSEγ concentration after immunoprecipitation, with the retention time of NSEγ indicated in the dashed-line box.*

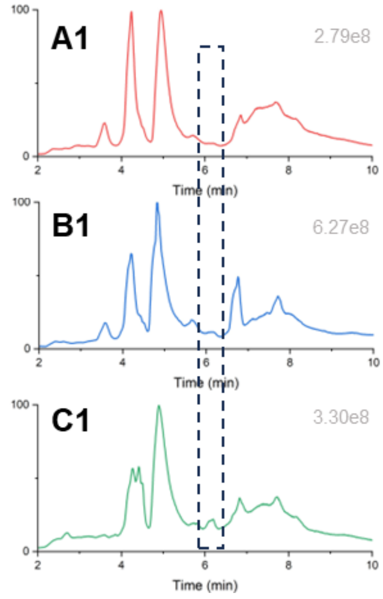

Other peaks observed are linked to non-specific protein binding to the magnetic beads. Peaks detected at respectively 3.58, 4.20, 4.83 and 6.76 min contained proteins with masses of respectively, 50641.0, 61439.0, 66438.3 and 28079.6 Da (Figure S2-Figure S5).

Figure S2*: Chromatogram (A1) Charge state envelope (A2) and deconvoluted intact mass (A3) of co-immunoprecipitating protein at tr: 3.58 min.*

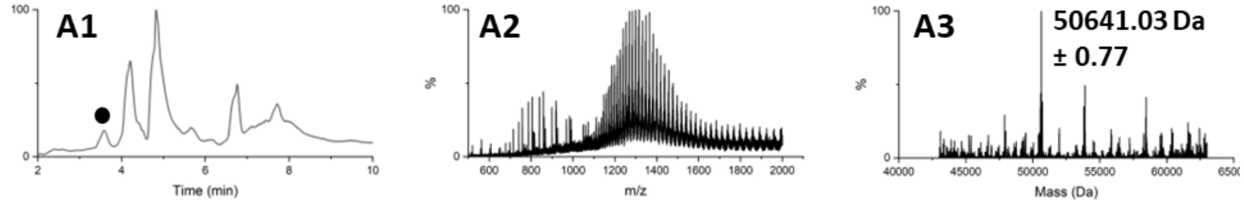

Figure S3*: Chromatogram (A1) Charge state envelope (A2) and deconvoluted intact mass (A3) of co-immunoprecipitating protein at tr: 4.20 min.*

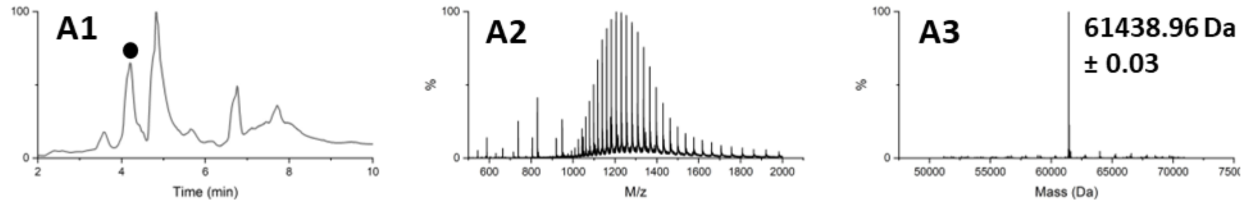

Figures S4*: Chromatogram (A1) Charge state envelope (A2) and deconvoluted intact mass (A3) of co-immunoprecipitating protein at tr: 4.83 min and is expected to be Humans Serum Albumin.*

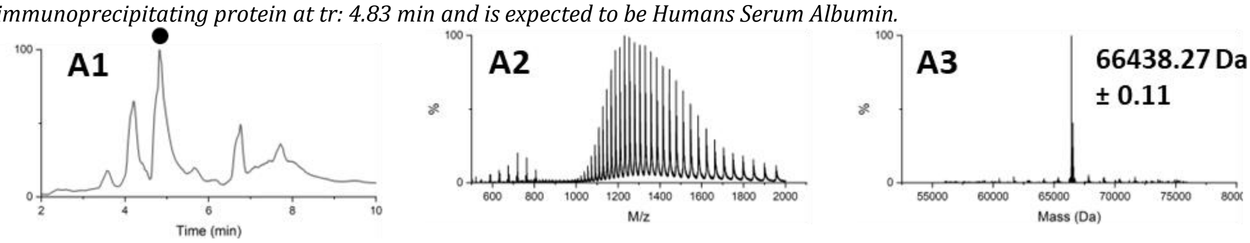

Figure S5: *Chromatogram (A1) Charge state envelope (A2) and deconvoluted intact mass (A3) of co-immunoprecipitating protein at tr: 6.76 min and is expected to be Apolipoprotein AI.*

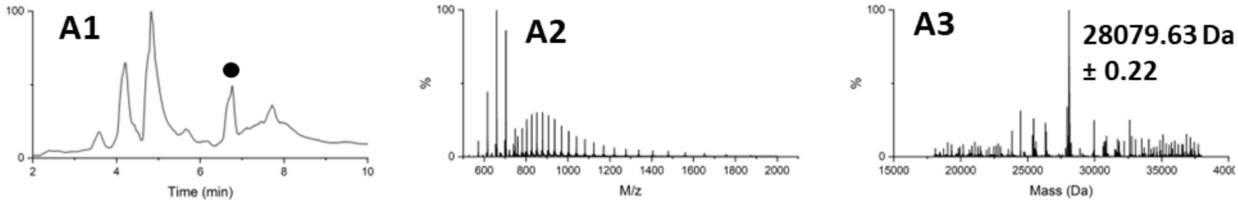

Supp Material S1: *Amino Acid Sequence Neuron Specific Enolase Gamma*

SIEKIWAREILDSRGNPTVEVDLYTAKGLFRAAVPSGASTGIYEALELRDGDKQRYLGKGVLKAVDHINSTIAPALISSGLSVVEQEK
LDNLMLELDGTENKSKFGANAILGVSLAVCKAGAAERELPLYRHIAQLAGNSDLILPVPAFNVINGGSHAGNKLAMQEFMILPVG
AESFRDAMRLGAEVYHTLKGVIKDKYGKDATNVGDEGGFAPNILENSEALELVKEAIDKAGYTEKIVIGMDVAASEFYRDGKYDL
DFKSPTDPSRYITGDQLGALYQDFVRDYPVVSIEDPFDQDDWAAWSKFTANVGIQIVGDDLTVTNPKRIERAVEEKACNCLLLKV
NQIGSVTEAIQACKLAQENGWGVMVSHRSGETEDTFIADLVVGLCTGQIKTGAPCRSERLAKYNQLMRIEEELGDEARFAGHNFR
NPSVL

Calculated Theoretical Mass: 47137.07 Dalton

Uniprot code P09104
